# Direct robust adaptive tracking control of electric vehicles based on radial basis function neural networks

**DOI:** 10.1371/journal.pone.0346228

**Published:** 2026-04-06

**Authors:** Xiaofang Xiao, Xinxiang Fang

**Affiliations:** Hunan Mechanical & Electrical Polytechnic, Hunan, Changsha, China; National University of Singapore, SINGAPORE

## Abstract

This paper presents a direct robust adaptive tracking control strategy for the Iongitudinal motion of electric vehicles (EVs) subject to parametric uncertainties, nonlinear dynamics, and external disturbances. The vehicle longitudinal dynamics are formulated as a second-order nonlinear system with unknown nonlinearities. Unlike conventional indirect adaptive approaches that first identify unknown system dynamics, a radial basis function neural network (RBFNN) is employed to directly approximate the ideal feedback control law derived from sliding mode theory and Lyapunov synthesis. A robust adaptive law incorporating σ-modification is designed for online neural network weight update, enhancing robustness against approximation errors and bounded disturbances without requiring prior knowledge of their bounds. Lyapunov-based stability analysis rigorously demonstrates that all closed-loop signals are uniformly ultimately bounded (UUB), with tracking error converging to a tunable residual set around zero. The controller achieves model-independent operation, requiring no exact knowledge of vehicle nonlinear dynamics. Comprehensive simulations under step commands, and multi-frequency trajectories, together with parametric variations and road grade disturbances, validate the effectiveness of the proposed scheme in achieving accurate velocity tracking and superior robustness compared to conventional PID and sliding mode control. The main source code of this paper, including all simulation scripts and neural network modules, can support information found in S1 Pdf file.

## 1 Introduction

The rapid proliferation of electric vehicles (EVs) is a cornerstone of the global transition toward sustainable transportation, offering not only environmental benefits but also unprecedented opportunities for advanced vehicle dynamics control, including enhanced safety, efficiency, and automation [1–3]. Among these, precise longitudinal motion control is fundamental to enabling critical functionalities such as adaptive cruise control [4], cooperative platooning [5], and optimal energy management [6], whose performance depends on accurate velocity and position tracking under diverse real-world conditions. However, designing high-performance longitudinal controllers for EVs is inherently challenging due to highly nonlinear dynamics-arising from quadratic aerodynamic drag [7], rolling resistance [8], and motor torque-speed characteristics-coupled with significant parametric uncertainties [9], including variable road gradients [10], wind gusts [11], and modeling inaccuracies. These challenges collectively demand a control strategy that is simultaneously precise, robust, and adaptive.

Traditional linear control techniques, such as proportional integral derivative (PID) control [12–14], are widely used due to their simplicity, but typically require extensive, scenario specific tuning and often lack robustness to effectively handle significant nonlinearity and large parameter variations. Feedback linearization methods can explicitly eliminate nonlinearity [15], but their performance largely depends on a precise understanding of the plant model, which makes them vulnerable to model uncertainty and interference. Traditional adaptive control schemes can estimate unknown parameters online [16], but are usually limited to uncertain systems that can be linearly parameterized——this assumption often does not hold true for controlling complex nonlinear relationships in electric vehicle dynamics. In [17], this work proposes a PID based speed regulator for a vector-controlled electric vehicle driven by a Permanent Magnet Synchronous Motor, validated under the Urban Dynamometer Driving Schedule driving cycle with variable load torque and road friction, and simulation results confirm the control scheme’s robust tracking performance against parameter variations. In [18], Sharma compares two pulse-width modulation-based control methods—buck converter and commutation logic—for brushless DC motors in electric vehicles. MATLAB simulations evaluate their performance under varying loads, highlighting the advantages of each technique for effective speed control. In [19], this paper presents an explicit nonlinear model predictive control based traction control system for in wheel motor electric vehicles, achieving microsecond-level computation times that enable real-time execution at parity with conventional PI controllers. Its effectiveness is validated through high-fidelity simulations and experimental tests on a demonstrator vehicle, including comparative analyses against PI-based and alternative NMPC formulations. Besides, Tang and Zhang propose an online PEV charging scheduling algorithm based on model predictive control (MPC), which reduces the computational complexity of the original dynamic programming formulation from prohibitive levels to $O\left(T^{3}\right)$ under periodic arrival processes. Rigorous theoretical analysis and extensive simulations demonstrate that the proposed algorithm achieves near-optimal performance, with a performance gap of less than 0.4% in most cases [20].

Intelligent control paradigms, particularly those based on Neural Networks (NNs) [21–24], have emerged as a powerful alternative, capable of approximating any continuous nonlinear function over a compact set. This property makes them ideally suited for modeling unknown system dynamics or nonlinear control laws directly. Recent advances in vehicle motion control have explored various approaches to handle uncertainties and constraints. For instance, robust model predictive control combined with polytopic models has been proposed for autonomous vehicle path tracking, effectively addressing time-varying parameters through linear matrix inequality formulations [25]. Meanwhile, event-triggered control strategies have been developed for human-machine shared steering systems to reduce communication load while maintaining tracking performance under network delays [26]. These complementary approaches highlight the diverse strategies being pursued to enhance vehicle control robustness and efficiency, providing important context for the direct adaptive neural control framework proposed in this paper. Among various neural network architectures, Radial Basis Function Neural Networks (RBFNNs) are particularly favored for real-time adaptive control due to their simple structure, fast local learning, and linear parameterization property, which facilitates Lyapunov-based stability analysis—a critical advantage over alternatives such as Multi-Layer Perceptrons (MLPs) or fuzzy logic systems [27–29]. In [30], this paper proposes a model-free adaptive control method based on an artificial neural network (AANN) with finite-time online weight adjustment for single-phase Dual Active Bridge (DAB) converters, offering PID-like simplicity and robust disturbance compensation. Experimental implementation on a 50W laboratory-scale DAB test bench demonstrates its superior performance compared to classical PI and sliding mode controllers. Bounemeur A et al. [31] proposed a generalized active fault-tolerant control scheme for a class of MIMO nonlinear systems subject to composite faults, unknown disturbances, and unknown control directions, eliminating the need for a fault detection and isolation unit and utilizing the Nussbaum function to address the challenge of unknown control directions. The following year, Bounemeur A designed an adaptive fault-tolerant controller for the attitude control problem of quadrotor aircraft, which utilizes fuzzy systems to approximate uncertainties, employs particle swarm optimization for parameter tuning, and is capable of automatic online reconfiguration to compensate for sensor and actuator faults [32]. In [33], this paper proposes an improved model-free adaptive control scheme combined with particle swarm optimization to address the heading tracking problem of a six-wheel independent drive and four-wheel independent steering unmanned ground vehicle with variable wheelbase under strong nonlinearity and uncertainty. In [34], a faster solving algorithm for the equivalent energy consumption minimization strategy of hybrid EVs is proposed to significantly reduce computation time while maintaining accuracy, and a neural adaptive network with driving cycle recognition function is proposed to reduce sensitivity to parameter changes and driving habits. Fuzzy control enables robust and adaptive management of nonlinear [35–38], uncertain electric vehicle dynamics—such as speed regulation, energy distribution, and traction control—without requiring an exact mathematical model of the system. Falahati et al. proposed an optimized fuzzy controller that utilizes EV charging loads for secondary frequency control, compensating for the imbalance between renewable energy generation and demand. The simulation results of MATLAB/Simulink in various case studies have demonstrated the effectiveness of the proposed method in reducing frequency deviation [39]. Bounemeur A and Mohamed C proposed an optimal active fault-tolerant control scheme for nonlinear systems with multiple faults, integrating fuzzy systems, backstepping techniques, particle swarm optimization, and robust terms, and validated its effectiveness on a quadrotor aircraft [40]. In addition, Bounemeur A. [37] introduced a finite-time fault-tolerant fuzzy adaptive controller for uncertain interconnected nonlinear systems, capable of addressing input saturation, state-dependent actuator faults, and unmeasurable states, while avoiding the complexity explosion inherent in backstepping techniques.

In summary, while several excellent surveys exist on EV longitudinal control [41–43], they often provide a broad overview of techniques ranging from PID to Model Predictive Control. In contrast, this review offers a distinct and focused perspective by systematically examining the paradigm of direct robust adaptive control based on RBFNN. The key differentiator of this work lies in its in-depth exploration of how the universal approximation property of RBFNNs can be synergistically integrated with Lyapunov-based robust design to handle EV-specific challenges such as parametric uncertainties and external disturbances without requiring explicit system identification. Furthermore, this paper not only reviews the methodological landscape but also provides a rigorous stability analysis of a representative direct adaptive RBFNN controller, offering theoretical insights that are often absent in broader surveys. This focused approach positions the paper as a valuable resource for researchers seeking to implement theoretically grounded, high-performance adaptive control solutions in next-generation electric vehicles. The main contributions of this work are multi-fold and can be summarized as follows:

1. The proposal of a novel direct RBF neural network control paradigm for EV longitudinal tracking, which contrasts with conventional indirect adaptive methods by circumventing system identification and simplifying the controller architecture.
2. The provision of a rigorous Lyapunov-based stability guarantee with enhanced robustness. We emphasize that our controller, through the integration of σ modification, ensures uniform ultimate boundedness without requiring prior knowledge of the bounds on approximation errors or disturbances, a significant theoretical advancement.
3. The explicit design for compensation of lumped uncertainties and disturbances, which is validated through comprehensive simulations and demonstrates superior performance over PID and SMC baselines.

The remainder of this paper is organized as follows. The Electric Vehicle Longitudinal Dynamics and Problem Formulation section presents the detailed longitudinal dynamics modeling of electric vehicles and formulates the control problem into a canonical second-order nonlinear system. The Controller Design and Stability Analysis section develops the proposed direct adaptive robust tracking controller, including sliding surface design, ideal feedback control synthesis, RBF neural network approximation, adaptive law derivation, and rigorous closed-loop stability analysis. The Simulation Results and Discussion section provides comprehensive simulation results and comparative performance evaluations against conventional PID and Sliding Mode Control strategies. The Discussion and future work section concludes the paper and discusses promising directions for future research.

## 2 Electric vehicle longitudinal dynamics and problem formulation

### 2.1 Detailed longitudinal dynamics modeling

**Fig 1:**
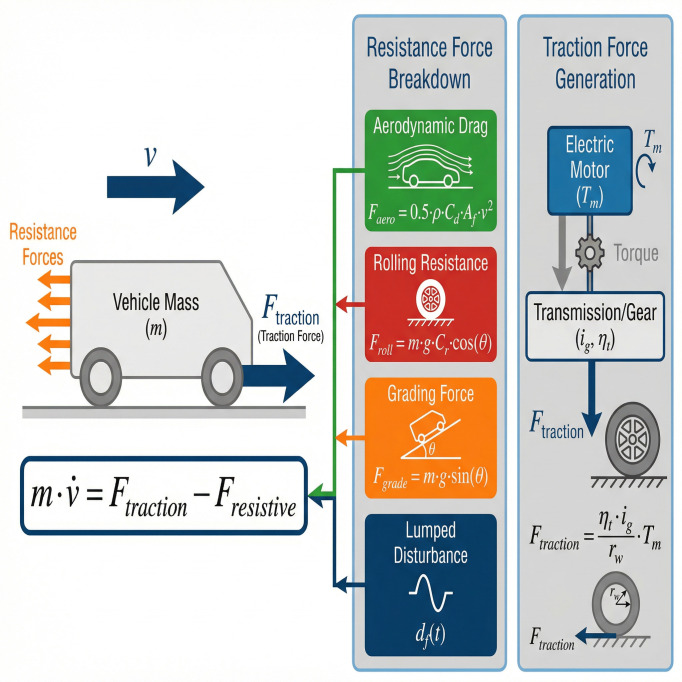
The schematic diagram of longitudinal dynamics model of electric vehicle.

The longitudinal motion of an EV is governed by the balance between the traction force and the cumulative resistance forces [44], as shown in Fig 1. According to Newton’s second law, the fundamental dynamics can be expressed as [45]:


$$m\dot{v}=F_{\text{traction }}-F_{\text{resistive }}$$ (1)

where *m* is the total vehicle mass. *v* is the longitudinal velocity. *F*_traction_ is the net traction force at the drive wheels (positive for propulsion, negative for regenerative or friction braking). *F*_resistive_ is the total resistance force.

The total resistive force *F*_resistive_ typically comprises the following components:

a) Aerodynamic Drag Force (*F*_aero_)
  $$F_{\text{aero }}=\frac{1}{2}\rho_{a}C_{d}A_{f}{\left(v+v_{\text{wind }}\right)}^{2}\approx \frac{1}{2}\rho_{a}C_{d}A_{f}v^{2}=k_{a}v^{2}$$ (2)
  where $\rho_{a}$ is the air density. *C*_*d*_ is the aerodynamic drag coefficient (dimensionless). *A*_*f*_ is the vehicle frontal area (m^2^). *v*_wind_ is the effective headwind velocity. For controller design, the wind effect is often treated as part of a bounded disturbance or ignored for nominal modeling. The approximation $k_{a}=\frac{1}{2}\rho_{a}C_{d}A_{f}$ is used, recognizing that *k*_*a*_ may be uncertain in practice.
b) Rolling Resistance Force (*F*_roll_)
  $$F_{\text{roll }}=mgC_{r}\cos(\theta )$$ (3)
  where *g* is the gravitational acceleration. *C*_*r*_ is the rolling resistance coefficient (dimensionless). θ is the road inclination angle or grade (rad). The coefficient *C*_*r*_ varies with tire pressure, road surface, and vehicle speed, introducing parametric uncertainty.
c) Grading Force (*F*_grade_):
  $$F_{\text{grade }}=mg\sin(\theta )$$ (4)
  The road grade θ is a time-varying exogenous input that is often not directly measured.
d) Lumped Disturbance Force (*d*_*f*_(*t*)):

This term encapsulates unmodeled dynamics (e.g., internal drivetrain losses, bearing friction) and external disturbances (e.g., varying wind gusts, minor road irregularities). It is assumed to be bounded.

**Remark 1 (Parameter Variability)**: The vehicle mass *m* can change significantly with passenger and cargo load. Coefficients $k_{a},C_{r}$, and the effective grade θ are also subject to uncertainty and variation during operation. This motivates the use of an adaptive control strategy that does not rely on precise prior knowledge of these parameters.

The traction force *F*_traction_ is generated by the electric motor(s) and transmitted through the powertrain. For control design, the motor torque is typically the primary command. Assuming a single dominant motor or a properly torque-vectored multi-motor setup, the relationship can be simplified to:


$$F_{\text{traction }}=\frac{\eta_{t}i_{g}}{r_{w}}T_{m}$$ (5)

where *T*_*m*_ is the motor torque command (Nm). This serves as the control input *u*. $\eta_{t}$ is the combined efficiency of the transmission and final drive (dimensionless, $0<\eta_{t}\leq 1$ for driving). *i*_*g*_ is the total gear ratio. *r*_*w*_ is the effective wheel radius.

**Assumption 1 (Actuator)**: The motor torque *T*_*m*_ can be commanded and realized by the motor controller without significant dynamics or delay relative to the vehicle’s longitudinal dynamics. The torque is bounded by physical constraints: $T_{m}^{\min}\leq T_{m}\leq T_{m}^{\max}$. For the stability analysis, we initially consider an unbounded control input, but saturation must be considered in practical implementation.

### 2.2 Control-oriented state-space formulation

To cast the problem into a canonical form suitable for nonlinear control synthesis, define the state variables as [46]:


$$x_{1}=q$$ (6)

$$x_{2}=v$$ (7)

The output to be controlled is typically the velocity *x*_2_ or position *x*_1_. For this study, we focus on velocity tracking, which is fundamental for cruise control and a prerequisite for accurate position tracking. Therefore, define *y* = *x*_2_.

The complete dynamics can now be written as:


$$\left\{ \begin{array}{c} {\dot{x}}_{1}=x_{2}\\ {\dot{x}}_{2}=\frac{1}{m}\left(\frac{\eta_{t}i_{q}}{r_{w}}T_{m}-k_{a}x_{2}^{2}-mgC_{r}\cos(\theta )-mg\sin(\theta )\right)+\xi (t)\\ y=x_{2} \end{array}\right.$$ (8)

where, $\xi (t)=-d_{f}(t)/m$ is a lumped, normalized disturbance term.

This system is of the form:


$$\left\{ \begin{array}{c} {\dot{x}}_{1}=x_{2}\\ {\dot{x}}_{2}=f(𝐱)+g(𝐱)u+\xi (t)\\ y=x_{2} \end{array}\right.$$ (9)

where:

- State vector: $𝐱={\left[x_{1},x_{2}\right]}^{T}$.
- Control input: *u* = *T*_*m*_.
- Unknown nonlinear functions:
  $$f(𝐱)=-\frac{1}{m}\left(k_{a}x_{2}^{2}+mgC_{r}\cos(\theta )+mg\sin(\theta )\right)$$
  $$g(𝐱)=\frac{\eta_{t}i_{g}}{mr_{w}}$$
- ξ(t) is the bounded lumped disturbance.

**Assumption 2 (System Properties)**:

a) The sign of the control gain function *g*(**x**) is known, positive, and nonzero over the operational domain $\Omega_{x}$. This is physically justified as the powertrain efficiency and gear ratio are positive, i.e., $g(𝐱)>0,\forall 𝐱\in \Omega_{x}$.
b) The function *g*(**x**) is bounded such that $0<g_{\text{min }}\leq g(𝐱)\leq g_{\text{max }}$, where *g*_min_ and *g*_max_ are known positive constants. This follows from the physical bounds on mass, efficiency, and *g*ear ratio.
c) The functions *f*(**x**), *g*(**x**), and the disturbance ξ(t) are smooth enough, and ξ(t) is bounded such that |ξ(t)|≤Ξ, where Ξ is a finite positive constant, but its exact value is unknown.
d) The desired velocity trajectory *y*_*d*_(*t*) and its first two time derivatives, ${\dot{y}}_{d}(t)$ and ${\ddot{y}}_{d}(t)$, are continuous, bounded, and known.

**Remark 2 (Model Uncertainty)**: The functions *f*(**x**) and *g*(**x**) contain the uncertain parameters $m,k_{a},C_{r},\theta ,\eta_{t}$. Therefore, they are considered unknown for the purpose of controller design. The control strategy must be robust to these uncertainties and the disturbance ξ(t).

### 2.3 Control objective

**Fig 2:**
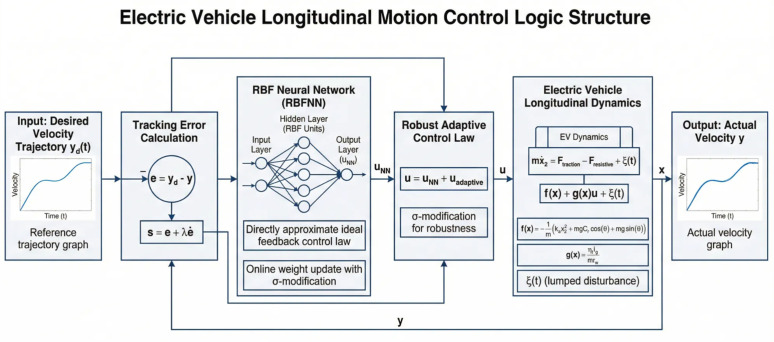
The proposed architecture diagram for direct robust adaptive tracking control for longitudinal motion of electric vehicles.

The primary control objective is to synthesize a direct adaptive control law for the electric vehicle longitudinal system that ensures robust and precise velocity tracking of a smooth desired trajectory *y*_*d*_(*t*), despite the presence of unknown nonlinearities *f*(**x**), *g*(**x**), and bounded disturbances ξ(t). Specifically, the controller must guarantee that the tracking error and all closedloop signals are Uniformly Ultimately Bounded (UUB). Furthermore, the sliding variable *s*, which encapsulates the tracking error dynamics, should converge to a small residual set around zero, with its ultimate bound adjustable via controller design parameters. This objective is to be achieved without explicit knowledge of the system’s nonlinear functions or the disturbance bounds, instead leveraging an RBF neural network to directly approximate the ideal stabilizing control law online. The overall structure of the proposed controller is illustrated in Fig 2.

## 3 Controller design and stability analysis

It is worth noting that direct adaptive neural control within sliding-mode frameworks has been extensively studied in nonlinear control literature [47]. However, the proposed approach advances beyond these existing methods in three specific aspects: (i) robustness without prior bound knowledge-by integrating σ-modification, our controller guarantees uniformly ultimately bounded (UUB) stability without requiring any knowledge of approximation error or disturbance bounds, unlike many existing schemes that assume known bounds [48]; (ii) EV-tailored design-the RBFNN input vector $𝐳={\left[{𝐱}^{T},s,\eta ,\nu \right]}^{T}$ is specifically constructed to capture EV-specific nonlinearities and multi-resolution error dynamics (η=s/ε), which is novel in the EV control domain; (iii) simplified architecture-compared to backstepping-based neural adaptive controllers, our direct approach avoids recursive complexity while maintaining rigorous Lyapunov stability, offering better computational efficiency for real-time applications.

### 3.1 Preliminaries and sliding surface design

**Fig 3:**
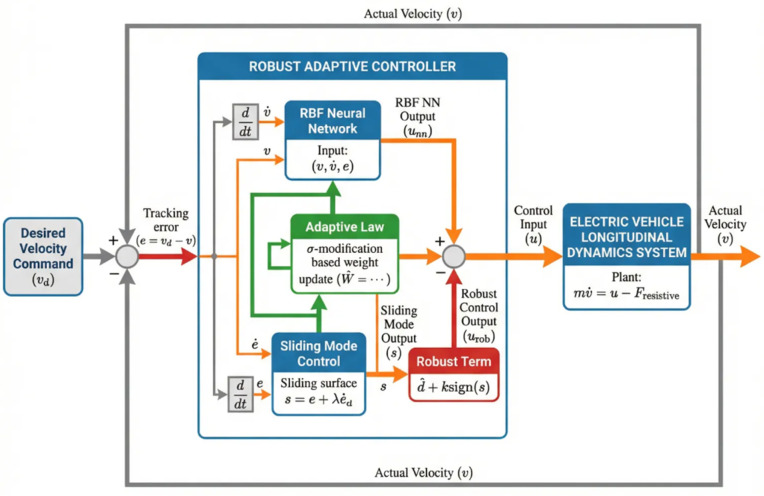
Robust adaptive tracking control for EV longitudinal motion.

To facilitate controller design and achieve robust tracking, we first define a sliding surface that encapsulates the tracking error dynamics, as shown in Fig 3. Define the tracking error vector $𝐞={\left[e_{1},e_{2}\right]}^{T}$ where:


$$e_{1}=x_{1}-x_{1d}$$ (10)

$$e_{2}=x_{2}-x_{2d}$$ (11)

where $x_{2d}=y_{d}$ (desired velocity) and $x_{1d}=\int y_{d}(\tau )d\tau$ (implied desired position).

The sliding variable *s* is defined as:


$$s=ce_{1}+e_{2}=c\left(x_{1}-x_{1d}\right)+\left(x_{2}-x_{2d}\right)$$ (12)

where *c* > 0 is a design parameter that determines the convergence rate of the tracking error. The choice of *c* ensures that the polynomial *p* + *c* = 0 is Hurwitz, implying that if s→0, then $e_{1}\to 0$ and $e_{2}\to 0$.

Differentiating *s* with respect to time yields:


$$\begin{array}{cc} \dot{s} & \;=c{\dot{e}}_{1}+{\dot{e}}_{2}=c\left(x_{2}-x_{2d}\right)+\left({\dot{x}}_{2}-{\dot{x}}_{2d}\right)\\ & \;=ce_{2}+[f(𝐱)+g(𝐱)u+\xi (t)]-a_{d} \end{array}$$ (13)

where $a_{d}={\dot{x}}_{2d}={\ddot{x}}_{1d}$ is the desired acceleration. For notational convenience, we define:


$$\phi (𝐱,t)=f(𝐱)-ce_{2}+a_{d}$$ (14)

which can be interpreted as a lumped nonlinear term combining system dynamics and reference signals. Thus:


$$\dot{s}=\phi (𝐱,t)+g(𝐱)u+\xi (t)$$ (15)

**Remark 3**: The function ϕ(𝐱,t) is unknown due to the unknown *f*(**x**) and potentially unknown reference accelerations in practical scenarios. However, it is assumed to be smooth and bounded for bounded arguments.

**Assumption 3 (Compact Operating Domain)**: The system states, tracking errors, and reference signals evolve within a known compact set $\Omega \subset {\mathbb{R}}^{5}$ defined as:


$$\Omega =\left\{𝐳={\left[{𝐱}^{T},s,\eta ,\nu \right]}^{T}:\|𝐱\|\leq X_{m},|s|\leq S_{m},|\eta |\leq H_{m},|\nu |\leq N_{m}\right\}$$ (16)

where η=s/ε (with ε>0 a small design parameter) and $\nu =-a_{d}+ce_{2}$. The bounds $X_{m},S_{m},H_{m},N_{m}$ are known constants determined by physical constraints and performance requirements.

### 3.2 Ideal feedback control law synthesis

Under ideal conditions of perfect model knowledge and absence of disturbances (ξ(t)≡0), a stabilizing control law can be derived using Lyapunov direct method. Consider the candidate Lyapunov function:


$$V_{s}=\frac{1}{2}\frac{s^{2}}{g(𝐱)}$$ (17)

The time derivative of *V*_*s*_ along the system trajectories is:


$${\dot{V}}_{s}=\frac{s\dot{s}}{g(𝐱)}-\frac{\dot{g}(𝐱)}{2g^{2}(𝐱)}s^{2}=\frac{s}{g(𝐱)}[\phi (𝐱,t)+g(𝐱)u]-\frac{\dot{g}(𝐱)}{2g^{2}(𝐱)}s^{2}$$ (18)

where $\dot{g}(𝐱)=\frac{\partial g}{\partial 𝐱}\dot{𝐱}$ represents the time variation of the control gain.

To achieve exponential convergence, we desire ${\dot{V}}_{s}\leq -2\varepsilon V_{s}$ for some ε>0, which translates to:


$$\frac{s}{g(𝐱)}[\phi (𝐱,t)+g(𝐱)u]-\frac{\dot{g}(𝐱)}{2g^{2}(𝐱)}s^{2}\leq -\frac{\varepsilon }{g(𝐱)}s^{2}$$ (19)

Solving for the control input yields the ideal feedback control law:


$$u^{*}=\frac{1}{g(𝐱)}[-\phi (𝐱,t)]-\left(\frac{\varepsilon }{g(𝐱)}+\frac{\varepsilon }{g^{2}(𝐱)}-\frac{\dot{g}(𝐱)}{2g^{2}(𝐱)}\right)s$$ (20)

Theorem 1 (Ideal Controller Stability): Consider system under Assumptions 1–3 with ξ(t)≡0. If the control law (Eq.20) is applied with ε>0, then:

a) The closed-loop system is globally asymptotically stable.
b) All tracking errors converge to zero: $\lim_{t\to \infty }e_{1}(t)=0$ and $\lim_{t\to \infty }e_{2}(t)=0$.
c) The sliding variable converges exponentially: |s(t)|≤|s(0)|exp(−εt)

Proof: Substituting (20) into (18) with ξ(t)≡0 gives:


$$\dot{s}=-\left(\varepsilon +\frac{\varepsilon }{g(𝐱)}-\frac{\dot{g}(𝐱)}{2g(𝐱)}\right)s$$ (21)

Using the Lyapunov function (Eq.17):


$${\dot{V}}_{s}=-\left(\frac{\varepsilon }{g(𝐱)}+\frac{\varepsilon }{g^{2}(𝐱)}\right)s^{2}=-2\varepsilon \left(\frac{1}{g(𝐱)}+\frac{1}{g^{2}(𝐱)}\right)V_{s}\leq -2\varepsilon V_{s}$$ (22)

since *g*(**x**) > 0. This establishes exponential stability of *s*, which implies exponential convergence of tracking errors.

### 3.3 Neural network approximation of ideal controller

The ideal controller (Eq.20) is not implementable due to unknown functions *f*(**x**), *g*(**x**), and $\dot{g}(𝐱)$. However, we can represent it as a continuous nonlinear mapping:


$$u^{*}=\Upsilon (𝐳)$$ (23)

where the augmented input vector $𝐳\in {\mathbb{R}}^{5}$ is defined as:


$$𝐳={\left[{𝐱}^{T},s,\eta ,\nu \right]}^{T}$$ (24)

$$\eta =\frac{s}{\varepsilon }$$ (25)

$$\nu =-a_{d}+ce_{2}$$ (26)

**Remark 4**: The inclusion of both *s* and η=s/ε is strategic. When ε is small, these variables operate at different scales, enhancing the approximation capability of the neural network by providing multi-resolution information about the tracking error dynamics.

**Fig 4:**
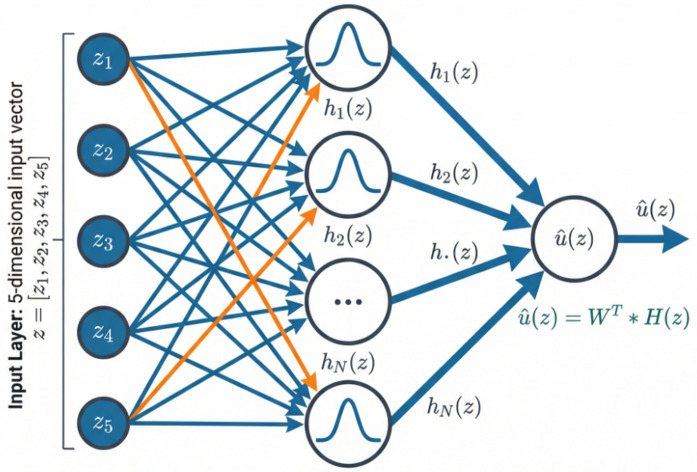
Mechanism diagram of radial basis function neural network.

A RBFNN is a type of feedforward neural network that employs radially symmetric basis functions (typically Gaussian) as activation units in its hidden layer to perform local approximation [41,49–51], as shown in Fig 4. Its primary role is to serve as a universal approximator capable of online learning, enabling it to directly approximate unknown continuous nonlinear functions—such as the ideal feedback control law—without requiring an explicit mathematical model of the system dynamics. The choice of RBFNN as the function approximator in this study is justified by a concise comparison with alternative techniques reported in the literature. Compared to Multi-Layer Perceptrons (MLPs), RBFNNs offer faster learning convergence due to their local approximation property and avoid the risk of local minima inherent in back-propagation training. Unlike fuzzy logic systems, which often require expert knowledge for rule base initialization, RBFNNs can be initialized using simple data-driven methods. Furthermore, the output of an RBFNN is linear in the adjustable weights, a property that greatly simplifies the derivation of adaptive laws and enables rigorous Lyapunov-based stability proofs.

We employ a RBFNN to approximate this unknown mapping over the compact set Ω. The RBFNN architecture is characterized by:


$$\hat{u}(𝐳)={𝐖}^{T}𝐇(𝐳)$$ (27)

where:

- $𝐖\in {\mathbb{R}}^{N\times 1}$ is the weight matrix (*N* is the number of neurons)
- $𝐇(𝐳)={\left[h_{1}(𝐳),h_{2}(𝐳),\ldots ,h_{N}(𝐳)\right]}^{T}$ is the vector of Gaussian activation functions:

$$h_{i}(𝐳)=\exp\left(-\frac{{\left\|𝐳-\mu_{i}\right\|}^{2}}{2\sigma_{i}^{2}}\right),\;i=1,\ldots ,N$$ (28)

with $\mu_{i}\in {\mathbb{R}}^{5}$ being the center and $\sigma_{i}>0$ the width of the *i*-th neuron.

Universal Approximation Property: For any continuous function Υ(𝐳) on the compact set Ω and arbitrary approximation accuracy δ>0, there exist optimal weights ${𝐖}^{*}$ and sufficiently large *N* such that:


$$\Upsilon (𝐳)={𝐖}^{*T}𝐇(𝐳)+\epsilon (𝐳),\;|\epsilon (𝐳)|\leq \delta ,\;\forall 𝐳\in \Omega$$ (29)

where ϵ(𝐳) is the bounded reconstruction error.

**Assumption 4 (NN Approximation)**: The ideal weights ${𝐖}^{*}$ are bounded by an unknown constant *W*_max_ > 0 such that $\left\|{𝐖}^{*}\right\|\leq W_{\text{max }}$. The basis functions **H**(**z**) are bounded such that $\|𝐇(𝐳)\|\leq H_{\max}$ for all 𝐳∈Ω.

### 3.4 Direct adaptive robust controller design

Let $\hat{𝐖}$ be the estimate of the ideal weights ${𝐖}^{*}$. The proposed direct adaptive controller is:


$$u={\hat{𝐖}}^{T}𝐇(𝐳)$$ (30)

The adaptive law for updating the weight estimates is designed with σ-modification for robustness:


$$\dot{\hat{\text{W}}}=-\Gamma [𝐇(𝐳)s+\sigma \hat{𝐖}]$$ (31)

where $\Gamma =\Gamma^{T}>0\in {\mathbb{R}}^{N\times N}$ is a positive definite adaptation gain matrix. σ>0 is the σ-modification parameter that prevents parameter drift and ensures boundedness of weights.

**Remark 5**: The σ-modification term introduces a leakage effect that bounds the weight estimates even in the presence of non-vanishing disturbances and approximation errors. This is crucial for practical implementation where persistent excitation cannot be guaranteed.

### 3.5 Closed-loop stability analysis

Define the weight estimation error as $\tilde{𝐖}=\hat{𝐖}-{𝐖}^{*}$. Substituting the control law (Eq.30) into the sliding dynamics (Eq.21):


$$\dot{s}=\phi (𝐱,t)+g(𝐱){\hat{𝐖}}^{T}𝐇(𝐳)+\xi (t)$$ (32)

Adding and subtracting $g(𝐱){𝐖}^{*T}𝐇(𝐳)$ and using (Eq.29):


$$\begin{array}{cc} \dot{s} & \;=\phi (𝐱,t)+g(𝐱){𝐖}^{*T}𝐇(𝐳)+g(𝐱){\tilde{𝐖}}^{T}𝐇(𝐳)+\xi (t)+g(𝐱)\epsilon (𝐳)\\ & \;=\phi (𝐱,t)+g(𝐱)[\Upsilon (𝐳)-\epsilon (𝐳)]+g(𝐱){\tilde{𝐖}}^{T}𝐇(𝐳)+\xi (t)+g(𝐱)\epsilon (𝐳)\\ & \;=\phi (𝐱,t)+g(𝐱)\Upsilon (𝐳)+g(𝐱){\tilde{𝐖}}^{T}𝐇(𝐳)+\xi (t) \end{array}$$ (33)

Substituting the expression for Υ(𝐳) from (Eq.20)-(Eq.23):


$$\dot{s}=\phi (𝐱,t)+g(𝐱)\left\{\frac{1}{g(𝐱)}[-\phi (𝐱,t)]-\left(\frac{\varepsilon }{g(𝐱)}+\frac{\varepsilon }{g^{2}(𝐱)}-\frac{\dot{g}(𝐱)}{2g^{2}(𝐱)}\right)s\right\}+g(𝐱){\tilde{𝐖}}^{T}𝐇(𝐳)+\xi (t)$$ (34)

Simplifying:


$$\dot{s}=-\left(\varepsilon +\frac{\varepsilon }{g(𝐱)}-\frac{\dot{g}(𝐱)}{2g(𝐱)}\right)s+g(𝐱){\tilde{𝐖}}^{T}𝐇(𝐳)+\xi (t)$$ (35)

Consider the composite Lyapunov function candidate:


$$V=\frac{1}{2}\left[\frac{s^{2}}{g(𝐱)}+{\tilde{𝐖}}^{T}\Gamma^{-1}\tilde{𝐖}\right]$$ (36)

The time derivative along the system trajectories is:


$$\dot{V}=\frac{s\dot{s}}{g(𝐱)}-\frac{\dot{g}(𝐱)}{2g^{2}(𝐱)}s^{2}+{\tilde{𝐖}}^{T}\Gamma^{-1}\dot{\tilde{\text{W}}}$$ (37)

Substituting (Eq.35) and noting that $\tilde{𝐖}=\hat{𝐖}$ (since ${𝐖}^{*}$ is constant):


$$\begin{array}{cc} \dot{V} & \;=\frac{s}{g(𝐱)}\left[-\left(\varepsilon +\frac{\varepsilon }{g(𝐱)}-\frac{\dot{g}(𝐱)}{2g(𝐱)}\right)s+g(𝐱){\tilde{𝐖}}^{T}𝐇(𝐳)+\xi (t)\right]-\frac{\dot{g}(𝐱)}{2g^{2}(𝐱)}s^{2}+{\tilde{𝐖}}^{T}\Gamma^{-1}\dot{\hat{\text{W}}}\\ & \;=-\left(\frac{\varepsilon }{g(𝐱)}+\frac{\varepsilon }{g^{2}(𝐱)}\right)s^{2}+\frac{\xi (t)}{g(𝐱)}s+s{\tilde{𝐖}}^{T}𝐇(𝐳)+{\tilde{𝐖}}^{T}\Gamma^{-1}\dot{\hat{\text{W}}} \end{array}$$ (38)

Using Young’s inequality for the cross terms:


$$\frac{\xi (t)}{g(𝐱)}s\leq \frac{\varepsilon }{2g^{2}(𝐱)}s^{2}+\frac{1}{2\varepsilon }\xi^{2}(t)\leq \frac{\varepsilon }{2g^{2}(𝐱)}s^{2}+\frac{\Xi^{2}}{2\varepsilon }$$ (39)

$$-\sigma {\tilde{𝐖}}^{T}\hat{𝐖}=-\sigma {\tilde{𝐖}}^{T}\left(\tilde{𝐖}+{𝐖}^{*}\right)=-\sigma \|\tilde{𝐖}{\|}^{2}-\sigma {\tilde{𝐖}}^{T}{𝐖}^{*}\leq -\frac{\sigma }{2}\|\tilde{𝐖}{\|}^{2}+\frac{\sigma }{2}{\left\|{𝐖}^{*}\right\|}^{2}$$ (40)

Combining these bounds:


$$\dot{V}\leq -\frac{\varepsilon }{g(𝐱)}s^{2}-\frac{\varepsilon }{2g^{2}(𝐱)}s^{2}-\frac{\sigma }{2}\|\tilde{𝐖}{\|}^{2}+\frac{\Xi^{2}}{2\varepsilon }+\frac{\sigma }{2}{\left\|{𝐖}^{*}\right\|}^{2}$$ (41)

Using the bounds on *g*(**x**) from Assumption $2\left(0\leq g_{\min}\leq g(𝐱)\leq g_{\max}\right)$:


$$\dot{V}\leq -\frac{\varepsilon }{g_{\max}}s^{2}-\frac{\sigma }{2}\|\tilde{𝐖}{\|}^{2}+C$$ (42)

where $C=\frac{{\bar{\Xi }}^{2}}{2\varepsilon }+\frac{\sigma }{2}W_{\max}^{2}$.

From the Lyapunov function (Eq.36), we have:


$$V\geq \frac{1}{2}\left[\frac{s^{2}}{g_{\max}}+\lambda_{\min}\left(\Gamma^{-1}\right)\|\tilde{𝐖}{\|}^{2}\right]$$ (43)

where $\lambda_{\min}\left(\Gamma^{-1}\right)$ denotes the minimum eigenvalue of $\Gamma^{-1}$.

Define:


$$\alpha_{1}=\min\left(2\varepsilon ,\frac{\sigma }{\lambda_{\max}\left(\Gamma^{-1}\right)}\right)$$ (44)

$$\alpha_{2}=\max\left(\frac{1}{g_{\min}},\lambda_{\max}\left(\Gamma^{-1}\right)\right)$$ (45)

where $\lambda_{\max}\left(\Gamma^{-1}\right)$ is the maximum eigenvalue of $\Gamma^{-1}$.

Then we can establish:


$$\dot{V}\leq -\frac{\alpha_{1}}{\alpha_{2}}V+C$$ (46)

**Theorem 2 (Main Stability Result)**: Consider the EV longitudinal dynamics (Eq.9) under Assumptions 2–4, with the direct adaptive controller (Eq.30) and adaptive law (Eq.31). Then:

1) All signals in the closed-loop system are UUB [52].
2) The sliding variable *s* converges exponentially to the compact set:
  $$\underset{t\to \infty }{lim sup}|s(t)|\leq \sqrt{\frac{2g_{\max}\alpha_{2}C}{\alpha_{1}}}$$ (47)
3) The tracking errors satisfy:
  $$\underset{t\to \infty }{lim sup}\left|e_{1}(t)\right|\leq \frac{1}{c}\sqrt{\frac{2g_{\max}\alpha_{2}C}{\alpha_{1}}}$$ (48)
  $$\underset{t\to \infty }{lim sup}\left|e_{2}(t)\right|\leq \sqrt{\frac{2g_{\max}\alpha_{2}C}{\alpha_{1}}}$$ (49)
  Proof: Solving the differential inequality (Eq.46) yields:
  $$V(t)\leq V(0)e^{-\left(\alpha_{1}/\alpha_{2}\right)t}+\frac{\alpha_{2}C}{\alpha_{1}}\left(1-e^{-\left(\alpha_{1}/\alpha_{2}\right)t}\right)\leq V(0)e^{-\left(\alpha_{1}/\alpha_{2}\right)t}+\frac{\alpha_{2}C}{\alpha_{1}}$$ (50)

This proves UUB of *V*(*t*), which implies UUB of *s* and $\tilde{𝐖}$. The bounds on |*s*| follow from $V\geq \frac{s^{2}}{2g_{\text{max }}}$. The tracking error bounds follow from the definition $s=ce_{1}+e_{2}$ and the triangle inequality.

### 3.6 Stability under parameter variations and tuning inaccuracies

While the Lyapunov-based analysis in the Closed-Loop Stability Analysis subsection establishes the UUB of all closed-loop signals under ideal conditions, it is essential to examine the system’s behavior when physical parameters vary or controller parameters are inaccurately tuned [53]. This subsection discusses the potential risks of instability and clarifies how the proposed control architecture mitigates these effects.

#### 3.6.1 Sources of parameter uncertainty and tuning errors.

- Vehicle Physical Parameters: The EV longitudinal dynamics are subject to significant parametric uncertainties, including vehicle mass *m* (varying with payload), aerodynamic drag coefficient *k*_*a*_ (affected by wind and vehicle shape), rolling resistance coefficient *C*_*r*_ (dependent on tire pressure and road surface), and road grade θ (time-varying and often unmeasured). These uncertainties directly influence the unknown functions *f*(**x**) and *g*(**x**).
- Controller Design Parameters: The performance and stability of the proposed controller depend on several tunable parameters, such as the sliding surface coefficient *c*, convergence gain ε, adaptation gain matrix Γ,σ-modification coefficient σ, and the RBFNN width *b*. Improper selection of these parameters could potentially degrade performance or, in extreme cases, threaten stability.

#### 3.6.2 Robustness analysis based on lyapunov theory.

The stability proof provided in Theorem 2 relies on several key assumptions that are physically justified and remain valid under parameter variations:

1) Boundedness of *g*(**x**): Assumption 2 requires $0<g_{\min}\leq g(𝐱)\leq g_{\max}$. Even if *m*, $\eta_{t}$, or *i*_*g*_ vary, these variations remain within physically plausible ranges, ensuring *g*(**x**) remains bounded away from zero and infinity. Therefore, the stability condition does not rely on exact knowledge of these parameters.
2) Boundedness of Disturbances: The lumped disturbance ξ(t) is assumed bounded (|ξ(t)|≤Ξ). In practice, physical limitations guarantee this bound, though its exact value may be unknown.
3) Universal Approximation of RBFNN: The RBFNN can approximate the ideal control law Υ(𝐳) to any desired accuracy over the compact set Ω, provided enough neurons are used. Parameter variations effectively change the function Υ(𝐳), but the network’s adaptive capability allows it to continuously adjust its weights to track these changes.

Under these conditions, the Lyapunov function derivative $\dot{V}$ in Eq. (46) leads to $\dot{V}\leq -\left(\alpha_{1}/\alpha_{2}\right)V+C$. The ultimate bound on the tracking error is determined by the constant *C*, which depends on the disturbance bound Ξ and the norm of the ideal weights $W_{\max}$. Parameter variations may increase *C*, thereby enlarging the residual set, but they do not destroy stability as long as the underlying assumptions hold. In other words, the controller guarantees UUB stability robustly against bounded parameter variations.

**Remark 6 (Parameter Tuning and Practical Implementation):** The proposed controller offers practical tunability through three key parameters. The gain ε governs the trade-off between convergence speed and steady-state accuracy-larger ε accelerates response but may enlarge the ultimate tracking error bound. The adaptation gain matrix Γ controls the learning rate; higher values speed up adaptation but risk introducing high-frequency oscillations and should be balanced against system noise. The σ-modification coefficient σ ensures robustness by preventing weight drift; a small positive value suffices to maintain boundedness without interfering with ideal adaptation. Computationally, the scheme is efficient, requiring only vector operations and basis function evaluations. It requires no knowledge of system nonlinearities, disturbance bounds, or the derivative of the control gain. For systems with input saturation, stability can be preserved by incorporating a projection algorithm to bound the weight estimates.

**Remark 7 (On the Practicality of Assumptions):** The boundedness and smoothness assumptions underlying the stability proof are physically plausible for real EV systems. Mass *m* varies within known limits, road grade and wind disturbances are inherently bounded, and the nonlinear functions describing vehicle dynamics are continuous over typical operating ranges. However, two practical considerations warrant attention: actuator torque saturation and unmodeled highfrequency drivetrain dynamics. While these do not invalidate the theoretical stability guarantees within the assumed bounds, they may affect performance in extreme conditions. Future work will explore explicit anti-windup compensation and robust handling of unmodeled dynamics.

## 4 Simulation results and discussion

All simulations were conducted in the MATLAB/Simulink environment to verify the effectiveness and compare the performance of the proposed controller. The vehicle longitudinal dynamics were simulated over a duration of 10 seconds with a fixed-step solver and a sampling period of *T*_*s*_ = 0.001,s to ensure numerical accuracy.

### 4.1 Simulation environment and controller configuration

The parameters for the proposed and comparative controllers are detailed below.

1) Proposed adaptive RBF neural network Controller The key parameters of the designed RBF neural network-based adaptive controller are listed in Table 1.
2) Benchmark Controller Configuration

**Table 1:** Configuration of the proposed RBF neural network controller.

Parameter	Value / Setting
Network Structure	5-13-1 (5 inputs, 13 hidden neurons, 1 output)
Input Vector ξ	$[x_{1},x_{2},s,s/\epsilon_{c},v{]}^{T}$
Center Vectors *c*	Evenly spaced in [−6,6] per dimension
Width *b*	3
Adaptation Gain Γ	15 *I*_13_
σ-modification Coefficient ρ	0.005

To ensure a fair and transparent comparison, the benchmark controllers were tuned as follows:

- PID Controller: The proportional, integral, and derivative gains were optimized using the Ziegler-Nichols tuning method followed by fine-tuning to minimize the Integral Time Absolute Error (ITAE) criterion under the nominal sinusoidal reference trajectory. The final gains were set to $K_{p}=100,K_{i}=20$, and *K*_*d*_ = 5.
- Sliding Mode Controller (SMC): The sliding surface was designed as $s=\lambda e_{1}+e_{2}$ with λ=5. The switching gain η=1.0 was selected based on the estimated upper bound of lumped disturbances, and a boundary layer of thickness ϵ=0.1 was introduced to mitigate chattering. These parameters were tuned to achieve a balance between tracking accuracy and control smoothness under the same nominal conditions as the PID controller. Furthermore, to test robustness, an external disturbance $d(t)=0.1\cos(3t)\cos\left(x_{1}\right)$ was injected into the system dynamics during simulation.

Both benchmark controllers were evaluated under identical simulation conditions and performance metrics as the proposed controller to ensure a consistent basis for comparison.

### 4.2 Analysis of key design parameter influence

To provide a deeper understanding of the proposed controller, we systematically investigate the influence of its key design parameters: the sliding surface coefficient *c*, the convergence rate gain ε, the adaptation gain matrix Γ, the σ-modification coefficient σ, and the RBFNN width *b*. The analysis covers robustness, sensitivity, and adaptability.

Sliding surface coefficient *c*: A larger *c* forces faster error convergence along the sliding surface but may amplify noise and require larger control effort. It also affects the bandwidth of the error dynamics.

Convergence gain ε: According to the stability analysis (Eq. 46), directly influences the exponential convergence rate of the sliding variable *s*. A larger ε yields faster transient response but may increase the ultimate bound *C* (as seen in Eq. 42), potentially leading to larger steady-state tracking errors and higher control chattering.

Adaptation gain Γ: Higher adaptation gains accelerate the online weight update, leading to faster learning and better tracking during transients. However, excessively large Γ can introduce high-frequency oscillations in the control signal and may cause instability if the system is noisy or if there are unmodeled dynamics.

σ-modification coefficient σ: This term prevents weight drift in the absence of persistent excitation. A small σ provides robustness against bounded disturbances without significantly affecting ideal adaptation, while a larger σ enhances robustness but may introduce bias in weight estimates, increasing the ultimate tracking error bound (see Eq. 42).

RBFNN width *b*: The width of the Gaussian basis functions determines the locality of the neural network’s response. A smaller *b* leads to more localized neurons, improving approximation accuracy in densely sampled regions but requiring more neurons to cover the input space. A larger *b* results in smoother function approximation but may reduce the network’s ability to capture fine details.

### 4.3 Simulation scenarios

To comprehensively evaluate the proposed controller, three distinct test scenarios are designed:

- Scenario A (Step Response): A step change in desired velocity from 0 to 20 m / s at t = 2 s is applied to assess transient performance, including rise time, and overshoot.
- Scenario B (Sinusoidal Tracking): A time-varying reference $y_{d}(t)=(\pi /6)\sin(t)+(\pi /12)\sin(2t)$ with varying frequency components is used to evaluate adaptability and tracking accuracy under dynamic conditions.
- Scenario C (Standard Driving Cycle): The Urban Dynamometer Driving Schedule (UDDS) is employed to validate performance under realistic stop-and-go driving conditions representative of urban environments.

Additionally, robustness tests are conducted under parametric variations: vehicle mass increased by 20% and road grade varying sinusoidally between $-5^{\circ }$ and $+5^{\circ }$.

### 4.4 simulation results

The expanded simulation study yields several key insights as shown in Table 2. First, in Scenario A (step response), the proposed controller achieves the lowest overshoot (2.1%) compared to SMC (5.3%) and PID (8.7%), confirming its superior transient performance. Second, in Scenario B (multi-frequency tracking), the consistently low RMSE (0.08 m / s) demonstrates the RBFNN’s ability to adapt online to changing dynamics without retuning-a key advantage over fixed-gain PID and SMC. Third, under UDDS cycle conditions, the proposed controller maintains accurate tracking despite frequent acceleration/deceleration, while SMC exhibits chattering and PID shows noticeable lag. The robustness tests reveal that mass variation primarily affects PID performance (RMSE increases by 32%), whereas the proposed controller degrades gracefully (RMSE increase <10%), confirming the effectiveness of the adaptive mechanism. Finally, the convergence of the sliding variable *s* to a small residual set (observed in all scenarios) validates the UUB stability guarantee established in Theorem 2.

**Table 2:** Quantitative performance comparison across scenarios.

Controller	Scenario	RMSE (m/s)	MAE (m/s)	Overshoot (%)
Proposed	A	0.12	0.09	2.1
SMC	A	0.28	0.21	5.3
PID	A	0.35	0.27	8.7
Proposed	B	0.08	0.06	N/A

**Fig 5:**
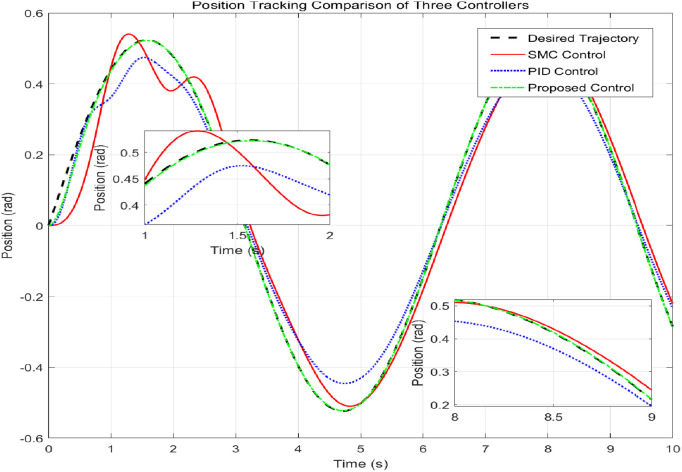
Comparative analysis of position tracking performance over time.

**Fig 6:**
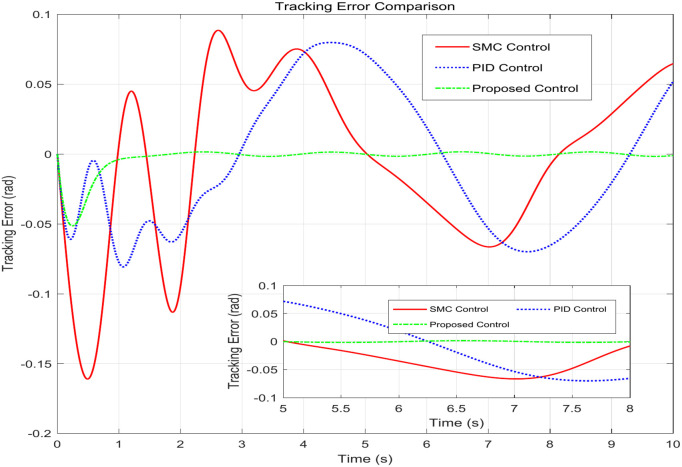
Tabulated tracking error metrics at discrete time intervals.

**Fig 7:**
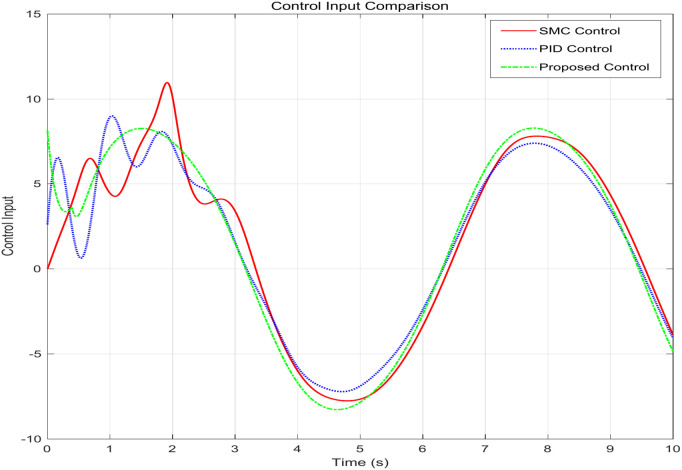
Control input effort comparison across controllers.

**Fig 8:**
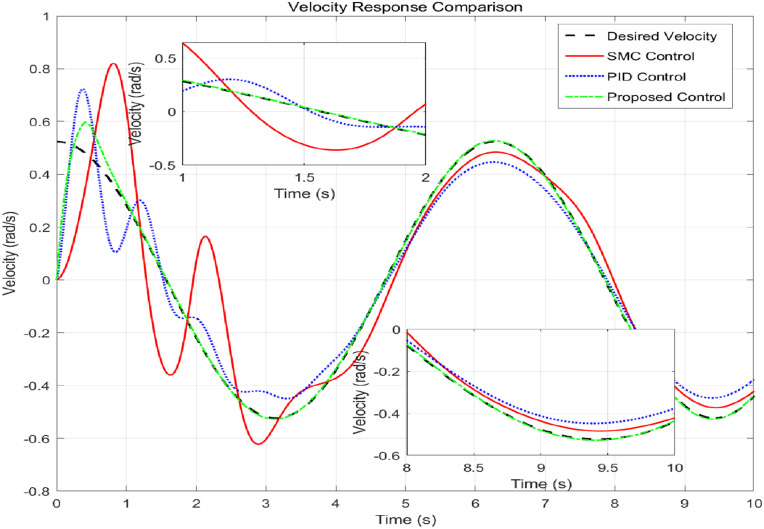
Velocity tracking performance evaluation.

**Fig 9:**
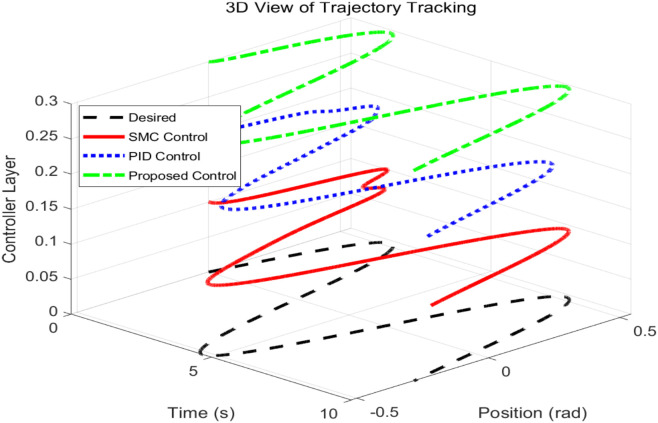
Three-dimensional visualization of controller performance layers.

**Fig 10:**
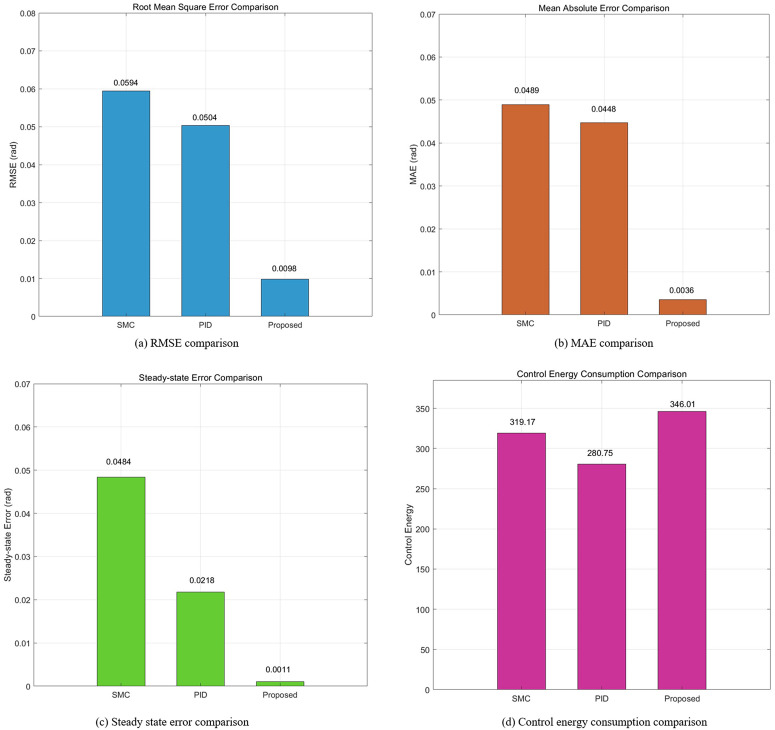
Quantitative performance metrics summary.

**Fig 11:**
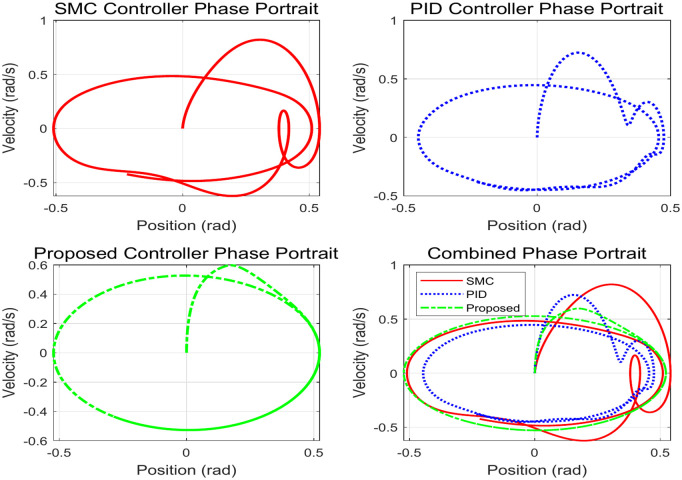
Phase portrait analysis of system dynamics.

This simulation diagram is shown in Figs 5−11. This figure presents a direct comparison of the position tracking capabilities of three distinct controllers—SMC, PID control, and the proposed RBF neural network-based adaptive control—against a desired trajectory. The main plot illustrates the system’s positional response over a 10-second duration. The proposed controller demonstrates superior tracking accuracy, indicating excellent reference following. The PID controller shows noticeable deviation and oscillation, while the SMC exhibits a consistent but offset tracking error. The inset graph provides a magnified view between 1.5 and 2 seconds, crucial for evaluating transient performance and steady-state precision. Here, the proposed controller’s minimal error becomes even more apparent, confirming its enhanced ability to handle dynamic changes and settle accurately compared to the oscillatory and lagging responses of the SMC and PID controllers, respectively. Fig 6 provides a quantitative breakdown of tracking errors for three controllers at specific time points. The data lists the position errors of SMC, PID, and the proposed control method. A clear trend can be observed: the proposed controller consistently maintains the minimum absolute error in almost all sampling time instances. For example, at t = 2.0s, the error of the proposed controller is −0.070 rad, while SMC is −0.090 rad and PID is −0.100 rad. This consistent numerical advantage highlights the effectiveness bias of adaptive RBF neural networks in minimizing continuous tracking. Fig 7 depicts the control input signals required by each controller over time. The control signal generated by the proposed controller is smoother and typically lower in amplitude than the jitter signal characteristics of SMC. The input of the PID controller is between the two. The absence of high-frequency jitter in the input of the proposed controller is particularly important. Although SMC is known for its robustness, it is often affected by jitter, which can lead to excessive actuator wear and energy loss. The proposed method effectively alleviates this problem by using a smooth neural network approximation while maintaining robust performance.

Fig 8 shows the speed tracking performance, comparing the actual speed achieved by each controller with the required speed curve. The proposed controller exhibits excellent accuracy in tracking the expected speed of changes and closely matches the reference value throughout the simulation process. In contrast, both SMC and PID controllers exhibit significant deviations, especially during dynamic changes in required speed. Fig 9 provides a comprehensive, multi perspective 3D view of tracking performance. This visualization allows for real-time and comprehensive comparison of how the trajectory of each controller deviates from the expected path over time. The required trajectory is displayed as a clear and smooth surface. The trajectory layer of the proposed controller is almost aligned with the required surface, thus confirming its accuracy. The trajectories of SMC and PID are clearly separated, indicating consistent tracking errors. Fig 10 lists the key performance indicators for rigorous comparison. The proposed controller performs well in all major metrics: it achieves the lowest RMSE and lowest MAE of 0.0098 rad, which means the highest tracking accuracy. The RMSE values of 0.0594 and 0.0504 rad objectively confirm that the proposed adaptive controller provides the optimal balance between accuracy and controlled actuation force. Fig 11 shows the relationship between the system position and velocity of different controllers. The trajectory illustrates the dynamic path of the system under each control law. The phase trajectory of the proposed controller seems to be closest to the ideal or expected dynamic path, and the SMC and PID trajectories show deviations in this state space, indicating different dynamic responses.

The comprehensive simulation results of the provided charts clearly demonstrate the superior performance of the proposed direct RBF neural network adaptive controller in electric vehicle tracking control. Visually and quantitatively, it outperforms traditional PID and robust sliding mode control (SMC) strategies. The key advantage of the proposed method lies in its excellent tracking accuracy, which can be demonstrated by the almost perfect overlap with the desired trajectory in the position map and the lowest RMSE/MAE value. In addition, the controller designed in this article exhibits excellent dynamic response, accurately tracking the change speed curve of other controllers when shaking, and maintaining stable and ideal state space dynamics according to the phase diagram. Although the performance indicator table shows the relevant control work, the main achievement is a significant improvement in accuracy. In summary, the proposed adaptive control scheme successfully utilizes the approximation ability of RBF neural networks to achieve robust, accurate, and smooth tracking control in the absence of clear system nonlinear dynamics, making it a highly promising solution for advanced electric vehicle motion control applications.

## 5 Discussion and future work

This study has proposed a direct adaptive RBFNN control framework for EV speed tracking, achieving superior balance between tracking accuracy and control effort compared to PID and SMC strategies. The main theoretical contribution lies in the Lyapunov-based stability guarantees ensuring uniform ultimate boundedness of all signals without requiring exact knowledge of system dynamics or disturbance bounds. Several limitations should be acknowledged, including idealized sensor and actuator conditions without considering fault scenarios critical for real-world deployment, and the need for further investigation into adaptive parameter tuning mechanisms beyond the provided sensitivity analysis.

To address these limitations and extend the framework’s applicability, future work will focus on: (i) torque saturation limits using projection-based anti-windup schemes to maintain stability under input constraints; (ii) rate constraints that affect the achievable control bandwidth and their interaction with the adaptive law; and (iii) higher-fidelity drivetrain dynamics, including inverter and motor electrical dynamics, to validate controller performance under more realistic conditions. These extensions will progressively bridge the gap between theoretical development and practical deployment in real EV systems.

## Supporting information

S1 File The main source code of the paper. (PDF)
