# Supplementary material for: Direct robust adaptive tracking control of electric vehicles based on radial basis function neural networks
Source: PLoS One. 2026 Apr 6;21(4):e0346228. doi: 10.1371/journal.pone.0346228 (PMC13052892; doi:10.1371/journal.pone.0346228)
Supplement: S1 File — (PDF) [file pone.0346228.s001.pdf]

```
%% Enhanced Comparative Simulation: RBF Neural Network vs. PID vs.
SMC
% -----------------------------------------------------------------------
% This script performs a comprehensive comparison of three controllers:
%   1. Proposed adaptive RBF neural network controller
%   2. Classical PID controller
%   3. Sliding mode controller (SMC) with boundary layer
%
% Features:
%   - Modular structure with separate functions for dynamics, controllers
%   - Multiple performance indices (RMSE, MAE, IAE, ISE, ITAE, ITSE,
etc.)
%   - Automatic figure generation with high-resolution export
%   - Multiple Monte-Carlo runs (to eliminate initialization randomness)
%   - Disturbance and reference trajectory configurability
%   - Full English comments, no Chinese characters
%
% Last revision: 2025
% -----------------------------------------------------------------------

clear; close all; clc;

%% ============================= 1. USER SETTINGS
============================

% ------------------------- Simulation time settings ----------------------
T_total = 10;              % Total simulation time [s]
Ts      = 0.001;           % Sampling period [s]
t       = 0:Ts:T_total;    % Time vector
N       = length(t);       % Number of simulation steps

% ------------------------- Desired trajectory ---------------------------
% Can be easily changed to test different references
yd_fun   = @(t) pi/6 * sin(t);        % Desired position
dyd_fun  = @(t) pi/6 * cos(t);        % Desired velocity
ddyd_fun = @(t) -pi/6 * sin(t);       % Desired acceleration

% ------------------------- External disturbance -------------------------
% d(t) = A * cos(omega * t) * cos(x1)   (state-dependent)
dist_amp   = 0.1;         % Amplitude
dist_omega = 3;           % Frequency

% ------------------------- Controller parameters -------------------------
% RBF neural network
```

```
rbf.lambda = 5;          % Sliding surface coefficient
rbf.epsilon= 0.25;       % Boundary layer thickness for input scaling
rbf.node   = 13;         % Number of hidden neurons
rbf.c      = 2 * repmat(linspace(-3,3,rbf.node), 5, 1);  % Centers (5x13)
rbf.b      = 3;          % Gaussian width
rbf.Gamma  = 15 * eye(rbf.node);   % Adaptation gain matrix
rbf.rho    = 0.005;      % σ-modification coefficient

% PID controller
pid.Kp = 100;   pid.Ki = 20;   pid.Kd = 5;

% Sliding mode controller
smc.lambda = 5;          % Sliding surface slope
smc.eta    = 1.0;        % Switching gain
smc.epsilon= 0.1;        % Boundary layer thickness

% ------------------------ Monte Carlo settings -------------------------
% Since RBF weights are initialized to zero, multiple runs are not strictly
% necessary. If random initialization is used, set num_runs > 1.
num_runs = 1;            % Number of independent simulation runs

% ------------------------ Figure export settings ----------------------
save_figures = true;     % Set to true to save all figures as PNG/PDF
fig_format   = {'-dpng', '-r300'};  % High-resolution PNG
fig_folder   = 'Simulation_Results'; % Folder to save figures

%% ============================= 2. INITIALIZATION
==========================
% Preallocate storage for multiple runs (if num_runs > 1)
% Only store the last run for visualization, but compute metrics averaged

% Cell arrays to store time series of each run
x1_rbf_all = cell(1,num_runs); x2_rbf_all = cell(1,num_runs);
u_rbf_all   = cell(1,num_runs); W_rbf_all   = cell(1,num_runs);

x1_pid_all = cell(1,num_runs); x2_pid_all = cell(1,num_runs);
u_pid_all   = cell(1,num_runs);

x1_smc_all = cell(1,num_runs); x2_smc_all = cell(1,num_runs);
u_smc_all   = cell(1,num_runs); s_smc_all   = cell(1,num_runs);

d_all       = cell(1,num_runs);
```

```
% Metrics matrices: rows = runs, columns = metrics (to be defined later)
metrics_RBF = []; metrics_PID = []; metrics_SMC = [];

%% ============================ 3. MAIN SIMULATION LOOP
(Monte Carlo) ====
fprintf('=====================================================\n'
);
fprintf('Starting %d simulation run(s)...\n', num_runs);
fprintf('=====================================================\n'
);

for run_idx = 1:num_runs
   fprintf('Run #%d/%d ...\n', run_idx, num_runs);

   % -------------------- Initialize state vectors --------------------
   % RBF
   x1_rbf = zeros(1,N); x2_rbf = zeros(1,N);
   u_rbf  = zeros(1,N);
   W_rbf  = zeros(rbf.node, N);
   W_rbf(:,1) = zeros(rbf.node, 1);  % Zero initial weights

   % PID
   x1_pid = zeros(1,N); x2_pid = zeros(1,N);
   u_pid  = zeros(1,N);
   e_pid  = zeros(1,N); de_pid = zeros(1,N);
   ie_pid = 0;

   % SMC
   x1_smc = zeros(1,N); x2_smc = zeros(1,N);
   u_smc  = zeros(1,N);
   s_smc  = zeros(1,N);

   % Common
   d_vec  = zeros(1,N);
   e_rbf  = zeros(1,N);
   e_smc  = zeros(1,N);

   % -------------------- Time marching (Euler) ----------------------
   for k = 1:N-1
      % --- Disturbance (identical for all controllers) ---
      d_vec(k) = dist_amp * cos(dist_omega * t(k)) * cos(x1_rbf(k));

      % --- 3.1 RBF Neural Network Controller ---
```

```
% Tracking error and sliding surface
e_rbf(k) = x1_rbf(k) - yd_fun(t(k));
de_rbf   = x2_rbf(k) - dyd_fun(t(k));
s        = rbf.lambda * e_rbf(k) + de_rbf;
v        = -ddyd_fun(t(k)) + rbf.lambda * de_rbf;

% RBF input vector (5-dimensional)
xi = [x1_rbf(k); x2_rbf(k); s; s/rbf.epsilon; v];

% Compute Gaussian basis function outputs
h = zeros(rbf.node, 1);
for j = 1:rbf.node
   h(j) = exp(-norm(xi - rbf.c(:,j))^2 / (2 * rbf.b^2));
end

% Control law
W = W_rbf(:,k);
u_rbf(k) = W' * h;

% Weight adaptation law (with σ-modification)
dW = -rbf.Gamma * (h * s + rbf.rho * W);
W_rbf(:,k+1) = W_rbf(:,k) + dW * Ts;

% System dynamics (RBF)
[dx1, dx2] = system_dynamics(x1_rbf(k), x2_rbf(k), u_rbf(k),
d_vec(k));
x1_rbf(k+1) = x1_rbf(k) + dx1 * Ts;
x2_rbf(k+1) = x2_rbf(k) + dx2 * Ts;

% --- 3.2 PID Controller ---
e_pid(k)   = x1_pid(k) - yd_fun(t(k));
de_pid(k)  = x2_pid(k) - dyd_fun(t(k));
ie_pid     = ie_pid + e_pid(k) * Ts;

u_pid(k) = -pid.Kp * e_pid(k) - pid.Ki * ie_pid - pid.Kd * de_pid(k);

% System dynamics (PID)
[dx1, dx2] = system_dynamics(x1_pid(k), x2_pid(k), u_pid(k),
d_vec(k));
x1_pid(k+1) = x1_pid(k) + dx1 * Ts;
x2_pid(k+1) = x2_pid(k) + dx2 * Ts;

% --- 3.3 Sliding Mode Controller ---
```

```
        e_smc(k)  = x1_smc(k) - yd_fun(t(k));
        de_smc    = x2_smc(k) - dyd_fun(t(k));
        s_smc(k)  = smc.lambda * e_smc(k) + de_smc;

        % Obtain current system parameters
        [~, ~, alfax, betax] = system_dynamics(x1_smc(k), x2_smc(k), 0, 0);

        % Equivalent control
        u_eq = (-alfax + ddyd_fun(t(k)) - smc.lambda * de_smc) / betax;

        % Switching control (saturation to avoid chattering)
        if abs(s_smc(k)) > smc.epsilon
            u_sw = -smc.eta * sign(s_smc(k)) / betax;
        else
            u_sw = -smc.eta * s_smc(k) / smc.epsilon / betax;
        end
        u_smc(k) = u_eq + u_sw;

        % System dynamics (SMC)
        [dx1, dx2] = system_dynamics(x1_smc(k), x2_smc(k), u_smc(k),
d_vec(k));
        x1_smc(k+1) = x1_smc(k) + dx1 * Ts;
        x2_smc(k+1) = x2_smc(k) + dx2 * Ts;
    end

    % --- Store final step disturbance (for plotting) ---
    d_vec(N) = dist_amp * cos(dist_omega * t(N)) * cos(x1_rbf(N));

    % --- Save trajectories of current run ---
    x1_rbf_all{run_idx} = x1_rbf; x2_rbf_all{run_idx} = x2_rbf;
    u_rbf_all{run_idx}   = u_rbf;   W_rbf_all{run_idx}   = W_rbf;

    x1_pid_all{run_idx} = x1_pid; x2_pid_all{run_idx} = x2_pid;
    u_pid_all{run_idx}   = u_pid;

    x1_smc_all{run_idx} = x1_smc; x2_smc_all{run_idx} = x2_smc;
    u_smc_all{run_idx}   = u_smc;   s_smc_all{run_idx}   = s_smc;

    d_all{run_idx}       = d_vec;

    % --- Compute performance metrics for this run ---
    yd_vec = yd_fun(t);
```

```
    m_RBF = compute_metrics(x1_rbf, yd_vec, u_rbf, Ts);
    m_PID = compute_metrics(x1_pid, yd_vec, u_pid, Ts);
    m_SMC = compute_metrics(x1_smc, yd_vec, u_smc, Ts);

    metrics_RBF = [metrics_RBF; m_RBF];
    metrics_PID = [metrics_PID; m_PID];
    metrics_SMC = [metrics_SMC; m_SMC];

    fprintf('   Run #%d completed.\n', run_idx);
end

%% ========================= 4. AVERAGE PERFORMANCE
========================
% Average metrics over all runs
avg_RBF = mean(metrics_RBF, 1);
avg_PID = mean(metrics_PID, 1);
avg_SMC = mean(metrics_SMC, 1);

% Standard deviation (if multiple runs)
std_RBF = std(metrics_RBF, 0, 1);
std_PID = std(metrics_PID, 0, 1);
std_SMC = std(metrics_SMC, 0, 1);

% For visualization, take the last run (or first if only one)
x1_rbf = x1_rbf_all{end}; x2_rbf = x2_rbf_all{end};
u_rbf  = u_rbf_all{end};   W_rbf  = W_rbf_all{end};

x1_pid = x1_pid_all{end}; x2_pid = x2_pid_all{end};
u_pid  = u_pid_all{end};

x1_smc = x1_smc_all{end}; x2_smc = x2_smc_all{end};
u_smc  = u_smc_all{end};   s_smc  = s_smc_all{end};

d_vec  = d_all{end};

%% ========================= 5. DISPLAY METRICS
============================
fprintf('\n=================================================\
n');
fprintf('PERFORMANCE COMPARISON (averaged over %d runs)\n',
num_runs);
fprintf('=================================================\n'
);
```

```
fprintf('Controller\tRMSE\t\tMAE\t\tIAE\t\tISE\t\tITAE\t\tITSE\t\tContr
olEnergy\n');
fprintf('RBF\t\t%.4f\t\t%.4f\t%.4f\t%.4f\t%.4f\t%.4f\t%.4f\n', avg_RBF);
fprintf('PID\t\t%.4f\t\t%.4f\t%.4f\t%.4f\t%.4f\t%.4f\t%.4f\n', avg_PID);
fprintf('SMC\t\t%.4f\t\t%.4f\t%.4f\t%.4f\t%.4f\t%.4f\t%.4f\n',  avg_SMC);
fprintf('====================================================\n'
);

%% ========================= 6. GENERATE FIGURES
===========================
% Desired trajectory vectors for plotting
yd_vec   = yd_fun(t);
dyd_vec  = dyd_fun(t);
ddyd_vec = ddyd_fun(t);

% Create output folder
if save_figures && ~exist(fig_folder, 'dir')
   mkdir(fig_folder);
end

% Figure 1: Position tracking
fig1 = figure('Name', 'PositionTracking', 'Position', [100 100 800 500]);
plot(t, yd_vec, 'k--', 'LineWidth', 2); hold on;
plot(t, x1_rbf, 'r-', 'LineWidth', 1.5);
plot(t, x1_pid, 'b:', 'LineWidth', 1.8);
plot(t, x1_smc, 'g-.', 'LineWidth', 1.5);
xlabel('Time (s)', 'FontSize', 12);
ylabel('Position (rad)', 'FontSize', 12);
title('Trajectory Tracking Comparison', 'FontSize', 14);
legend({'Desired', 'RBF', 'PID', 'SMC'}, 'Location', 'northeast', 'FontSize',
11);
grid on; box on; set(gca, 'FontSize', 11);
if save_figures
   export_figure(fig1, fig_folder, 'Fig1_Position_Tracking');
end

% Figure 2: Tracking error
fig2 = figure('Name', 'TrackingError', 'Position', [150 150 800 500]);
plot(t, x1_rbf - yd_vec, 'r-', 'LineWidth', 1.5); hold on;
plot(t, x1_pid - yd_vec, 'b:', 'LineWidth', 1.8);
plot(t, x1_smc - yd_vec, 'g-.', 'LineWidth', 1.5);
xlabel('Time (s)', 'FontSize', 12);
ylabel('Error (rad)', 'FontSize', 12);
```

```
title('Tracking Error Comparison', 'FontSize', 14);
legend({'RBF', 'PID', 'SMC'}, 'Location', 'northeast', 'FontSize', 11);
grid on; box on; set(gca, 'FontSize', 11);
if save_figures
    export_figure(fig2, fig_folder, 'Fig2_Tracking_Error');
end

% Figure 3: Control inputs
fig3 = figure('Name', 'ControlInput', 'Position', [200 200 800 500]);
plot(t(1:end-1), u_rbf(1:end-1), 'r-', 'LineWidth', 1.5); hold on;
plot(t(1:end-1), u_pid(1:end-1), 'b:', 'LineWidth', 1.8);
plot(t(1:end-1), u_smc(1:end-1), 'g-.', 'LineWidth', 1.5);
xlabel('Time (s)', 'FontSize', 12);
ylabel('Control Input', 'FontSize', 12);
title('Control Effort Comparison', 'FontSize', 14);
legend({'RBF', 'PID', 'SMC'}, 'Location', 'northeast', 'FontSize', 11);
grid on; box on; set(gca, 'FontSize', 11);
if save_figures
    export_figure(fig3, fig_folder, 'Fig3_Control_Input');
end

% Figure 4: Velocity tracking
fig4 = figure('Name', 'VelocityTracking', 'Position', [250 250 800 500]);
plot(t, dyd_vec, 'k--', 'LineWidth', 2); hold on;
plot(t, x2_rbf, 'r-', 'LineWidth', 1.5);
plot(t, x2_pid, 'b:', 'LineWidth', 1.8);
plot(t, x2_smc, 'g-.', 'LineWidth', 1.5);
xlabel('Time (s)', 'FontSize', 12);
ylabel('Velocity (rad/s)', 'FontSize', 12);
title('Velocity Response Comparison', 'FontSize', 14);
legend({'Desired', 'RBF', 'PID', 'SMC'}, 'Location', 'northeast', 'FontSize',
11);
grid on; box on; set(gca, 'FontSize', 11);
if save_figures
    export_figure(fig4, fig_folder, 'Fig4_Velocity_Tracking');
end

% Figure 5: Sliding surface of SMC
fig5 = figure('Name', 'SlidingSurface', 'Position', [300 300 800 400]);
plot(t, s_smc, 'b-', 'LineWidth', 1.5);
xlabel('Time (s)', 'FontSize', 12);
ylabel('Sliding surface s', 'FontSize', 12);
title('Sliding Surface (SMC)', 'FontSize', 14);
```

```
grid on; box on; set(gca, 'FontSize', 11);
if save_figures
    export_figure(fig5, fig_folder, 'Fig5_Sliding_Surface');
end

% Figure 6: RBF weight norm evolution
fig6 = figure('Name', 'RBFWeightNorm', 'Position', [350 350 800 400]);
W_norm = sqrt(sum(W_rbf.^2, 1));
plot(t, W_norm, 'r-', 'LineWidth', 1.5);
xlabel('Time (s)', 'FontSize', 12);
ylabel('||W||', 'FontSize', 12);
title('RBF Neural Network Weight Norm', 'FontSize', 14);
grid on; box on; set(gca, 'FontSize', 11);
if save_figures
    export_figure(fig6, fig_folder, 'Fig6_RBF_Weight_Norm');
end

% Figure 7: External disturbance
fig7 = figure('Name', 'Disturbance', 'Position', [400 400 800 400]);
plot(t, d_vec, 'm-', 'LineWidth', 1.5);
xlabel('Time (s)', 'FontSize', 12);
ylabel('d(t)', 'FontSize', 12);
title('External Disturbance', 'FontSize', 14);
grid on; box on; set(gca, 'FontSize', 11);
if save_figures
    export_figure(fig7, fig_folder, 'Fig7_Disturbance');
end

% Figure 8: Phase portrait comparison (combined)
fig8 = figure('Name', 'PhasePortrait', 'Position', [450 450 800 600]);
subplot(2,2,1);
plot(x1_rbf, x2_rbf, 'r-', 'LineWidth', 1.5);
xlabel('x_1 (rad)'); ylabel('x_2 (rad/s)');
title('RBF Phase Portrait'); grid on; box on;
subplot(2,2,2);
plot(x1_pid, x2_pid, 'b:', 'LineWidth', 1.8);
xlabel('x_1 (rad)'); ylabel('x_2 (rad/s)');
title('PID Phase Portrait'); grid on; box on;
subplot(2,2,3);
plot(x1_smc, x2_smc, 'g-.', 'LineWidth', 1.5);
xlabel('x_1 (rad)'); ylabel('x_2 (rad/s)');
title('SMC Phase Portrait'); grid on; box on;
subplot(2,2,4);
```

```
plot(x1_rbf, x2_rbf, 'r-', 'LineWidth', 1.5); hold on;
plot(x1_pid, x2_pid, 'b:', 'LineWidth', 1.8);
plot(x1_smc, x2_smc, 'g-.', 'LineWidth', 1.5);
xlabel('x_1 (rad)'); ylabel('x_2 (rad/s)');
title('Combined Phase Portrait');
legend('RBF','PID','SMC','Location','best');
grid on; box on;
if save_figures
   export_figure(fig8, fig_folder, 'Fig8_Phase_Portrait');
end

% Figure 9: RMSE bar chart
fig9 = figure('Name', 'RMSE', 'Position', [500 500 600 450]);
rmse_vals = [avg_RBF(1), avg_PID(1), avg_SMC(1)];
bar(rmse_vals, 0.6, 'FaceColor', [0.3 0.6 0.8]);
set(gca, 'XTickLabel', {'RBF', 'PID', 'SMC'});
ylabel('RMSE (rad)', 'FontSize', 12);
title('Root Mean Square Error', 'FontSize', 14);
grid on; box on; set(gca, 'FontSize', 11);
% Add value labels
for i = 1:3
   text(i, rmse_vals(i)+0.0005, sprintf('%.4f', rmse_vals(i)), ...
      'HorizontalAlignment', 'center', 'FontSize', 10);
end
if save_figures
   export_figure(fig9, fig_folder, 'Fig9_RMSE');
end

% Figure 10: Control energy bar chart
fig10 = figure('Name', 'ControlEnergy', 'Position', [550 550 600 450]);
energy_vals = [avg_RBF(7), avg_PID(7), avg_SMC(7)];
bar(energy_vals, 0.6, 'FaceColor', [0.8 0.4 0.2]);
set(gca, 'XTickLabel', {'RBF', 'PID', 'SMC'});
ylabel('Control Energy', 'FontSize', 12);
title('Control Effort Comparison', 'FontSize', 14);
grid on; box on; set(gca, 'FontSize', 11);
for i = 1:3
   text(i, energy_vals(i)+0.5, sprintf('%.2f', energy_vals(i)), ...
      'HorizontalAlignment', 'center', 'FontSize', 10);
end
if save_figures
   export_figure(fig10, fig_folder, 'Fig10_Control_Energy');
end
```

```
% Figure 11: Error histogram (distribution of tracking error)
fig11 = figure('Name', 'ErrorHistogram', 'Position', [600 600 800 400]);
subplot(1,3,1);
histogram(x1_rbf - yd_vec, 50, 'FaceColor', 'r', 'EdgeColor', 'none');
xlabel('Error (rad)'); ylabel('Frequency'); title('RBF Error Distribution');
grid on; box on;
subplot(1,3,2);
histogram(x1_pid - yd_vec, 50, 'FaceColor', 'b', 'EdgeColor', 'none');
xlabel('Error (rad)'); ylabel('Frequency'); title('PID Error Distribution');
grid on; box on;
subplot(1,3,3);
histogram(x1_smc - yd_vec, 50, 'FaceColor', 'g', 'EdgeColor', 'none');
xlabel('Error (rad)'); ylabel('Frequency'); title('SMC Error Distribution');
grid on; box on;
if save_figures
   export_figure(fig11, fig_folder, 'Fig11_Error_Histogram');
end

% Figure 12: Comprehensive performance radar chart (optional, requires
extra toolbox)
% Skip if no radar chart function

fprintf('\nAll figures generated. Total figures: %d\n',
length(findall(0,'Type','figure')));

%% ======================== 7. SAVE WORKSPACE
============================
if save_figures
   save(fullfile(fig_folder, 'simulation_workspace.mat'), ...
       't','yd_vec','dyd_vec','ddyd_vec', ...
       'x1_rbf','x2_rbf','u_rbf','W_rbf', ...
       'x1_pid','x2_pid','u_pid', ...
       'x1_smc','x2_smc','u_smc','s_smc', ...

'd_vec','avg_RBF','avg_PID','avg_SMC','std_RBF','std_PID','std_SMC');
   fprintf('Workspace saved to %s\n', fullfile(fig_folder,
'simulation_workspace.mat'));
end

fprintf('\n=============================================\
n');
fprintf('SIMULATION COMPLETED SUCCESSFULLY.\n');
```

```
fprintf('==========================================================\n'
);

%% ============================= 8. AUXILIARY FUNCTIONS
===========================

function [dx1, dx2, alfax, betax] = system_dynamics(x1, x2, u, d)
   % System dynamics of the nonlinear plant
   % Inputs:
   %   x1, x2 : state variables
   %   u      : control input
   %   d      : external disturbance
   % Outputs:
   %   dx1, dx2 : state derivatives
   %   alfax, betax : system nonlinear functions (used by SMC)

   a1 = 0.5 * sin(x1) * (1 + cos(x1)) * x2^2 - 10 * sin(x1) * (1 + cos(x1));
   a2 = 0.25 * (2 + cos(x1))^2;
   alfax = a1 / a2;
   betax = 1 / a2;   % since b(x) = a2
   dx1 = x2;
   dx2 = alfax + betax * u + d;
end

% ----------------------------------------------------------------------
function m = compute_metrics(x1, yd, u, Ts)
   % Compute a comprehensive set of performance indices
   % Inputs:
   %   x1 : actual position trajectory
   %   yd : desired position trajectory
   %   u  : control input signal
   %   Ts : sampling period
   % Outputs: row vector with metrics
   %   [RMSE, MAE, IAE, ISE, ITAE, ITSE, ControlEnergy]

   e = x1 - yd;
   t_vec = (0:length(e)-1) * Ts;

   RMSE = sqrt(mean(e.^2));
   MAE  = mean(abs(e));
   IAE  = sum(abs(e)) * Ts;
   ISE  = sum(e.^2) * Ts;
   ITAE = sum(abs(e) .* t_vec) * Ts;
```

```
    ITSE = sum(e.^2 .* t_vec) * Ts;

    % Control energy (2-norm squared)
    ControlEnergy = sum(u(1:end-1).^2) * Ts;

    m = [RMSE, MAE, IAE, ISE, ITAE, ITSE, ControlEnergy];
end

% ---------------------------------------------------------------------
function export_figure(fig, folder, filename)
    % Export figure as PNG and PDF with high resolution
    png_path = fullfile(folder, [filename '.png']);
    pdf_path = fullfile(folder, [filename '.pdf']);

    % Set figure paper size and orientation for PDF
    set(fig, 'PaperPositionMode', 'auto');

    % Save PNG
    print(fig, png_path, '-dpng', '-r300');

    % Save PDF
    print(fig, pdf_path, '-dpdf', '-r600', '-bestfit');

    % Also save as EPS (optional, for LaTeX)
    % eps_path = fullfile(folder, [filename '.eps']);
    % print(fig, eps_path, '-depsc');
end
```
